# Protoplasm.—Its Relation to Vitality

**Published:** 1874-07

**Authors:** H. R. Noel

**Affiliations:** Profesor of Physiology.


### AMERICAN JOURNAL

OF

### DENTAL SCIENCE.

Vol. VIII. THIRD SERIES--JULY, 1874. No. 3.

### ARTICLE I.

### *Protoplasm—Its Relation to Vitality.*—Continued.

BY H. R. NOEL, M. D., PROFESSOR OF PHYSIOLOGY.

Valedictory Address delivered February 26th, 1874, at Baltimore College of Dental Surgery.

*Animal World.*—We turn now to the animal, and we seek the solution of the new problem of protoplasmic activity in that kingdom to which man belongs.

Has vitality in the animal a different meaning from vitality in the plant? Has "vital force" a different significance, and are its laws of manifestation in any respect different? What are the conditions of simply life in man? To eat, to drink, to breathe, to be kept warm. Food—water, oxygen—light and heat!

Eliminating oxygen or air from the discussion for a few minutes, we will consider the other conditions, and find that they are the same for the plant and for the animal; and the agent which manipulates these conditions is also the same; then the phenomenon of growth and development in the two are identical, or so very nearly so, that any radical distinction between them is not at present possible. And we are justified in the conclusion that whatever is predicable of protoplasmic activity in the one, is predicable of it in the other.

A critical analysis of "vitality" as shown in the vegetable kingdom, as already given us "construction," as the type of such life, where force *dynamic* becomes force *static*; what then is a vast forest but the *storehouse* of the *past?* And the venerable tree of a thousand years' growth, one which has lived through the rise and fall of nations, empires, dynasties, has through that long period been steadily but quietly preserving the light and heat of the sun, and will one day return, yes, inevitably return again to the dynamic form, all the force which for so many years it has held in the static.

The very essence then of "*vitality*" is the accumulation of material and the accumulation of force; and from the sprouting acorn to its culmination in the towering oak, the steps present one unbroken continuation of accretion, aggregation, growth. Then, if this be the history of vitality in plants, and we share with plants this *vitality*, it has a similar history in us, and the analysis of "vital force" in animals and in man must show that same accumulation of material and accumulation of static force.

*The Egg.*—There is an experiment in nature, which is so beautiful in its pure simplicity, so instructive in its scientific history, so attractive in its synthesis, and yet so perfect a demonstration of the identity of vitality in the two kingdoms, that I can not resist the temptation to give you a short account of it—an *epitome* it is—"'Tis only the hatching of an egg?" You may smile at so apparently plain a thing, yet this contains the key-note, the very arch and key-stone, and we shall be unwise builders if we neglect it

An egg—analyzed it is albumen and yolk, with *some* masses of protoplasm; the white and yolk are material, the protoplasm is of course the worker, mason, the carpenter, the architect, the builder; these protoplasmic agents remain quiescent until stimulated by heat, and then they immediately begin to work, and if the heat be steadily applied, they will in from 14 days to 30 days consume all of the material and a large amount of the heat, and in the place of the white and yolk there will be a living animal, a bird.

Will you be so kind as to recall the elements of this equation, and to retain them as I repeat them. Material+protoplasm+21 days' heat=one living animal. Now there is here no obscurity, no vague and untenable hypothesis, no visionary theories, but simply facts of observation, facts which philosophers can not misunderstand, and which physiologists can not but explain clearly; the white (albumenoid,) the yellow (oleaginous,) and the masses of protoplasm under continued heat have disappeared, and in their places a living animal is given us. There is and can be but one explanation of this change, and it is the plain statement that the protoplasmic agents have gone to work, and consumed the material in the manufacture of this animal, and they have also consumed the force called heat. But as heat is a force it is indestructible and continuous, and hence the "vitality" shown in the manufacture of this bird, being also a form of force, I must assume that the physical has by correlation become a *vital force*. Pardon the repetition, but I cannot too warmly or too earnestly commend to your thoughtful consideration this intensely interesting and lucid experiment of *animal building*. The statements are so few, the chance of misconception so small, and the result so perfect, that one cannot but recognize the same force, same conditions, same laws of action, and *same* final result, running in a linked series through both kingdoms; and if in the oak and all other species of vegetable productions, the essence of "vital force or protoplasmic action" be the accumulation of material and the accumulation of force, then will its essence be the same in the "*animal kingdom*," and an animal is but the expression of re-arranged *material* and *static* force. The re-arranged material we find in the feathers, flesh, blood and bones of the bird, and we here accept the evident change of form; but the heat which has been consumed, and which re-appeared as "vital force," and which has played so important a part in animal building, is it indeed rendered static? Is any heat or any form of force stored up with the material which we call the flesh? The answer to this question is easy indeed; the bird has a muscular system, nervous system, and a brain, and the very hour it emerges fram the shell, it gives us evidence of muscular force and nerve force; it runs and it cries. Now this "muscular and nerve force" which is *hatched with the bird*, must of course have been in a *static condition* in the system of the bird prior to hatching, and if that be true, as of necessity it must be, then we can trace the ascending "form of *heat*" through "*vital force*" into *static animal force.*

Now the animal forces are, muscular force, nerve force, and brain force, and at the very dawn of active animal existence, we find those forces ready in the animal, and they have only to be changed in form from the static to dynamic.

Force is stored up in muscle, it is stored up in nerve centres, it is stored up in that great nerve centre, the brain; and moreover it has been stored there by protoplasmic activity. In plainer language, the muscle is built and force is put in it; the brain is built and force is put in it, and the evolution of these forces from their static condition is animal life. All *animal force* depends therefore upon a prior existing "protoplasmic activity," called vital or vegetable force, and we can also conclude that the animal forces are directly as the pre-existing "vital force," which primarily constructs the tissues from which are evolved the animal forces.

*The Change from Static to Dynamic.*—A very pertinent inquiry put at this stage, is in what manner is this static force evolved or made dynamic? Now in the liberating of light and heat, which are static in *coal*, *wood*, oils, &c, we must have chemical destruction, chemical change, before the light and heat are rendered dynamic; you can only get the *active* or *dynamic* form by breaking up the *material conditions* of the *static*. Now is this also true of muscle and brain? Do we only get muscular force by destruction of muscular tissue and brain force by destruction of brain tissue, just as we get light and heat by destruction (we mean chemical only,) of the combination which holds them static? I think the answer is undoubtedly in the affirmative; and brain force means brain destruction, muscular force is muscular destruction. Then all exhibitions of animal force are essentially destructive to the tissue, acting and muscular work means muscular waste, and brain work means brain waste; but how is this tissue destroyed in the functional activity of brain and muscle?

We burn coal to get heat and light: so we also burn brain and muscle to get intellectual action or brain force and motion or muscular force?

Again I answer in the affirmative, and now we see at least ONE very important use of oxygen which we breathe in the air; though oxygen is also useful in other combustions going on in the animal system. Atmospheric air contains 79 nitrogen and 21 oxygen, and we (each one,) fill and empty the lungs 17 times in a minute; we use about 2½ to 3 lbs. of oxygen every day, and we use 800 lbs. in a year; we use also 800 lbs. of food in a year, and hence we see that while food is evidently for constructive use, the oxygen is for destructive; one is therefore as essential as the other. A man then has two kinds of life, the first one essentially constructive and storing up; second one essentially destructive and liberating.

Man is in one sense a furnace, and the faster he burns the more force he sets free, but the supply of muscular and brain tissue is limited, and if burned too fast, we burn out too soon. It is fortunate for us that those protoplasmic agents which primarily construct our muscles, nerves, brain, and every other tissue and structure of the body, do not leave their work unguarded, but many are left to watch these tissues, and as they waste away these agents repair them. The blood furnishes an ever present supply of building material, and it also dissolves and carries off the burned elements as a soluble ash; the protoplasm obtains from the blood the materials with which to first construct, and second, maintain the integrity of the animal; so it not only builds, but it watches and incessantly repairs the waste.

From these propositions you at once conclude that *man* is a *machine*, which must be constructed before it is worked; and you also perceive the vast difference between the "*constructing agent*" and the machinery constructed. The agent builds the machinery and the process of building we call growth and development, but the working of this machinery is another thing, and this other thing is animal life. We have therefore a double life. 1st, vitality, eminently constructive and reparative. 2nd, animality, eminently destructive and wasting; and through life to death runs this same balance, or partial balance; from infancy to the 35th year the *constructive* is the predominant life; from 35th to 45th there is usually a *balance* of the two lives, and equalization of construction and destruction; but after the 45th the "constructive life begins to decline, the protoplasmic agents generally relax their activity, they build more slowly, they repair more slowly, and after the 70th or 80th year, they seem unwilling to work much longer, and man shrinks up, wastes away, and is not rebuilt, and hence the failure of animal forces as he floats slowly down the ebbing tide of vitality.

But you may ask what of this system working? What of this animality which is not vitality but is so absolutely dependant upon it? I can only say I did not promise to unfold the mysteries of "animal life," but only vegetative life; that undercurrent of life which precedes, determines, defines and measures the extent of the other.

*The Practical Application.*—There are some very curious applications of the theories above advocated, and yet some which are of every day occurrence in our practical lives; for if it be true that the protoplasmic activity is dependent upon material and dynamical conditions, which we call food and water, light and heat, then an inadequate supply of either must give decreased or perverted activity of the *builders;* hence a child with deficient food, deficient heat, deficient light, would assuredly sooner or later show evidence of inferior tissue building, if not of really decreased building; the protoplasmic agents having inferior material with which to build, will of course put up inferior structure, and we call this inferior structure a "scrofulous affair."

Again, suppose a man works his brain too severely and too long; suppose he does not take his 6 or 8 hours sleep each night, that the builders may during that time repair the day's waste; what do you expect as the ultimate result? He is burning his brain tissue faster than he is building it, and the end inevitable, the inexorable, is failure of brain force or aberration in its manifestation. Finally the man either *dies* of so-called "softening of the brain," or he glides downwards through the descending scale of irritability, eccentricity, incipient insanity, to fully developed loss of reason. And now the explosions of brain force are erratic, abnormal, irrational and we put him in a special institution called an "asylum," and give him good food and brain rest for 6 months or more, hoping that in that time the *builders* will repair the organ, and remove all traces of the trouble. Sometimes the builders of the lungs fail to do their work properly, and then we have a gradually increasing trouble in those organs, and the Doctor at once stops the man from work, gives him good food, plenty of sun-light and heat, plenty of sleep, and trusts to better building in future.

*Cancer.*—You have heard it said that "evil communications corrupt good manners;" this is true of the protoplasmic agents also, and we find now and then a species of insubordination, or extremely bad tissue building, beginning first in a localized manner, and then spreading over vast *areas*; take for example the rascally fraud in tissue building, which we call *cancer*. This is nearly invariably a local mutiny of the builders, and if the knife be freely used, and all of these disaffected agents promptly removed, the thing stops; but if one hesitates, delays, procrastinates for months, the mutiny spreads, and these protoplasmic agents get into the blood vessels, and then travel to other parts, and migrating out of the vessels, set up the same trouble elsewhere; then instead of one, we have many *foci* of trouble, and finally the man's whole system becomes involved and corrupted, and he dies of cancer. Dies because of the migratory habits of these protoplasmic agents, which being corrupted at the primary depot, have carried their acquired bad habits with them on their travels. Sometimes, in fact, in bone cancer, we find them stopping in the lungs, liver and other organs, and quietly building nodules of bone infected by cancer wherever they stop.

If there be a useful lesson to be learned from this essay, and it is "*the absolute necessity of a correct hygiene*, childhood is the period of constructive or protoplasmic activity in its most vigorous form, hence the demand for food, and hence the necessity for light and heat, and also for oxygen to supply the means of burning up the refuse matter. Therefore give your children good rich food, and plenty of air, sun-light, and good clothing. Again, beware of too much brain work in youth; too much schooling keeps the child too quiet, and he fails to get enough of light and oxygen, he therefore builds badly and sooner or later shows flagging. Beware of the cultivation of youthful prodigies, you are simply burning their brains up faster than they are repaired, and your prodigy of 4 or 6 years is the dunce 18 or 20; better first muscular development as a very gradual process. The builders of our body, like the builders of our houses, are only reliable when they work slowly, and rapid building in either case means defective work of uncertain strength and short duration. Therefore when in a case of typhoid fever, typhus fever, erysipelas, diptheria, &c., your physician orders beef tea, cream and rich milk, he at once assumes that your tissues are wasting faster than the protoplasmic workers can with your ordinary food effect repair; he also assumes that many of the workers have been killed, and yet from this decreased number he requires extra work, and to meet the emergency he furnishes the best of material, and in addition often orders wine and stimulants, which are said not only to arrest tissue destruction, but also to supply encouragement to the builders; and thus he endeavors to preserve enough of tissue to ward off a "collapse of animality" until the stress of the disease is over; and now more than ever recognizing the fact that in the integrity of the builders is the preservation of the structures upon which the evolution of animality so directly hinges.

In conclusion permit me to return in the name of the FACULTY of the College, our thanks for the compliment of your presence to night; and if, as its humble representative, I have succeeded in throwing some light upon the origin of that interesting subject, the "maintenance of life," and preservation of vital force, I shall be most happy to have discharged my duty, first to you in giving the true basis for a correct hygiene, and secondly to them as teachers in a school, which has for its object the preservation of certain organs almost indispensably necessary to a proper preparation of one of the conditions of life, namely—*food.*

---

